# Food and Nutrition Surveillance System (SISVAN) coverage, nutritional status of older adults and its relationship with social inequalities in Brazil, 2008-2019: an ecological time-series study

**DOI:** 10.1590/S2237-96222023000100003

**Published:** 2023-03-20

**Authors:** Brena Barreto Barbosa, Valéria Troncoso Baltar, Rogério Lessa Horta, Jackeline Christiane Pinto Lobato, Luiza Jane Eyre de Souza Vieira, Caroline de Oliveira Gallo, Antonio Augusto Ferreira Carioca

**Affiliations:** 1 Universidade Estadual do Ceará, Programa de Pós-Graduação em Nutrição e Saúde, Fortaleza, CE, Brasil; 2 Universidade Federal Fluminense, Departamento de Epidemiologia e Bioestatística, Niterói, RJ, Brasil; 3 Universidade FEEVALE, Mestrado Acadêmico em Psicologia, Novo Hamburgo, RS, Brasil; 4 Universidade de Fortaleza, Pós-Graduação em Saúde Coletiva. Fortaleza, CE, Brasil; 5 Universidade de São Paulo, Faculdade de Saúde Pública, São Paulo, SP, Brasil

**Keywords:** Food and Nutrition Surveillance, Older Adult Health, Overweight, Public Health Service Coverage, Social Inequality, vigilancia Nutricional, Salud del Anciano, Sobrepeso, Cobertura de Servicios Públicos de Salud, Factores Socioeconómicos, Vigilância Alimentar e Nutricional, Saúde do Idoso, Sobrepeso, Cobertura de Serviços Públicos de Saúde, Desigualdade Social

## Abstract

**Objective:**

to analyze the temporal trend of Food and Nutrition Surveillance System (*Sistema de Vigilância Alimentar e Nutricional* - SISVAN) coverage and the nutritional status of older adults, and its correlation with indicators of social inequality in Brazil between 2008-2019.

**Methods:**

this was an ecological study using records from SISVAN, related to the population aged 60 years and older; the temporal trend of coverage and the correlation between indicators of social inequality and increment rate of nutritional status were analyzed; slope index of inequality and concentration index were used to measure absolute and relative inequalities.

**Results:**

11,587,933 records were identified; national coverage increased from 0.1% (2008) to 2.9% (2019), with a statistically significant upward trend; a moderate inverse correlation with an annual increment rate of overweight between human development index and gross domestic product *per capita*, was found.

**Conclusion:**

there was an increasing trend in SISVAN coverage; the increase in overweight was associated with social inequality.

## Resumo

**Objetivo:**

analisar a tendência temporal da cobertura do Sistema de Vigilância Alimentar e Nutricional (SISVAN) e do estado nutricional de idosos, e sua correlação com indicadores de desigualdade social no Brasil, no período 2008-2019.

**Métodos:**

estudo ecológico, sobre registros do SISVAN relativos à população na idade de 60 anos ou mais; analisaram-se a tendência temporal da cobertura e a correlação entre indicadores de desigualdade social e taxa de incremento do estado nutricional; os índices angular e de concentração foram utilizados para medir desigualdades absolutas e relativas.

**Resultados:**

foram identificados 11.587.933 registros de idosos; a cobertura nacional evoluiu de 0,1% (2008) para 2,9% (2019), com tendência de aumento estatisticamente significativa; foi encontrada correlação inversa moderada com taxa de incremento anual de sobrepeso, para índice de desenvolvimento humano e produto interno bruto *per capita*.

**Conclusão:**

houve tendência de crescimento da cobertura do SISVAN; o aumento de sobrepeso esteve associado à desigualdade social.

## Resumen

**Objetivo:**

analizar la tendencia temporal de cobertura del Sistema de Vigilancia Alimentaria y Nutricional (SISVAN), y el estado nutricional de adultos mayores, correlacionándolos con indicadores de desigualdad social, en el período 2008-2019.

**Métodos:**

estudio ecológico mediante registros del SISVAN sobre la población ≥60 años. Se realizaron análisis de correlación entre indicadores de desigualdad social y la tasa de incremento del estado nutricional y análisis de desigualdades absolutas y relativas para obtener el índice de desigualdad angular y el índice de concentración.

**Resultados:**

se identificaron 11.587.933 registros. La cobertura nacional evolucionó del 0,1% en 2008 al 2,9% en 2019, con una tendencia ascendente estadísticamente significativa. Se encontró una correlación inversa moderada con la tasa de incremento anual de sobrepeso para IDH y PIB per cápita.

**Conclusión:**

hubo una tendencia de crecimiento en la cobertura del SISVAN. El aumento del sobrepeso se asoció con la desigualdad social.

**Study contributions**

**Main results**

The national coverage rose from 0.1% (2008) to 2.9% (2019), a significant upward trend. Moderate inverse correlation with annual increment rate of overweight between human development index and gross domestic product *per capita*, was found.

**Implications for services**

The low percentage of coverage results in insufficient data for the development and adjustment of public policies aimed at older adults. Regions with the worst social indicators may present a larger population of adults who are overweight, affecting health services.

**Perspectives**

The increase in the coverage of the nutritional status of older adults by SISVAN is essential for planning health actions. It can be seen the need to incorporate the actions of SISVAN into the routine Primary Health Care, as a way to boost its coverage.

## INTRODUCTION

Food and Nutrition Surveillance (*Vigilância Alimentar e Nutricional* - VAN) is one of the guidelines of the National Food and Nutrition Policy (*Política Nacional de Alimentação e Nutrição* - PNAN), and allows the description and prediction of trends in the food and nutritional status of the Brazilian population, aiming at health promotion. VAN is carried out through the Food and Nutrition Surveillance System (*Sistema de Vigilância Alimentar e Nutricional* - SISVAN), operated by the Primary Health Care (PHC) with the objective of monitoring the dietary pattern and nutritional status of users of the Brazilian National Health System (*Sistema Único de Saúde* - SUS).^1^ Created in 2008, the SISVAN online platform (SISVAN Web) has enabled the monitoring of the food consumption and nutritional status and the identification of population groups at risk for nutritional problems.^2^

Nutritional status monitoring, using data from SISVAN, is performed by calculating body mass index (BMI), based on anthropometric measurements (body weight; height) of SUS users from different population strata: preschoolers and students, adolescents, adults, pregnant women and older adults.^3^ However, the highest frequency of records in the system is for preschoolers, students, adolescents and pregnant women,^4^ due to the criteria of the former Family Income Transfer Program (*Programa Bolsa Família*), currently Programa Auxílio Brasil, which is the main source of information for SISVAN. These programs present, as one of the conditionalities in the health sector, the nutritional monitoring of children under 7 years of age and prenatal care for pregnant women, aimed at preventing or reducing problems such as malnutrition, childhood obesity and maternal and infant mortality.^5^

There have been positive changes in access to health services and in the reduction of socioeconomic inequalities in the country over the past 40 years, leading to a decrease in infant mortality and maternal mortality, partly attributed to conditional cash transfer programs, such as *Programa Bolsa Família*.^6^ The accelerated aging process of the Brazilian population, in recent decades, the consequent increase in life expectancy and, at the same time, the increase in the occurrence of chronic non-communicable diseases, leading causes of death and disability in the country, demanded greater health care to the health conditions of the older adult population.^7^

Brazil is among the most unequal countries in terms of economic and social status, which is one of the main determinants of malnutrition among the population. Inequality has worsened recently, as indicated by the increasing trend of the Gini index, from 0.506 in 2019 to 0.519 in 2022. The Gini index predicts outcomes on a scale of zero to 1, where numbers closer to zero indicate greater equality. These inequalities have been deepened as a result of the pandemic caused by the novel coronavirus, which started in 2020.^8^

The investigation and monitoring of the nutritional status of older adults is important for early identification of risk factors for nutritional problems, enabling adjustments to nutritional intervention measures, aiming at preventing or reducing damage to health in this population.^9^ However, the coverage of nutritional status by SISVAN has been lower in this age group,^4^ representing a factor that has contributed to worsening food security among older adults.

Knowledge on the coverage of nutritional status of older adults by SISVAN and its relationship with the indicators of social inequality is important for improving PNAN and monitoring food and nutrition indicators, based on data from the System.

Thus, the objective of this study was to analyze the temporal trend of SISVAN coverage and the nutritional status of older adults, correlating it with indicators of social inequality in Brazil between 2008 and 2019.

## METHODS

*Study design*

This was an ecological time-series study, based on secondary data available on SISVAN Web platform, for the period 2008 to 2019. The units of analysis corresponded to Brazil, its macro-regions (North; Northeast; South; Southeast; Midwest) and the Federative Units (FUs). Data were extracted from the System on December 21, 2020.

*Setting*

The first version of SISVAN was made available by the Ministry of Health in 2004. In 2008, its new platform, SISVAN Web, was released, and it is available on the internet. This new version allowed the registration and access to anthropometric assessment and food consumption information of the entire population receiving PHC services within the SUS.^2^ In 2017, SISVAN, version 3.0, was released, which optimized its integration with e-SUS Primary Care (e-SUS *Atenção Básica* - e-SUS AB) and it is remotely accessed (https://sisaps.saude.gov.br/sisvan/). Open access annual reports are available on SISVAN Web platform. They consolidate all types of follow-ups, registered by health professionals during the (VAN) actions in the PHC, and those carried out by e-SUS AB and the Bolsa Família Program Management System, which periodically, migrate automatically to SISVAN platform.^2^ Programa Auxílio Brasil replaced Bolsa Família Program in November 2021, maintaining its functionality as a source of information for SISVAN. This name change occurred after the data collection period of this research, which is why reference is made to Bolsa Família Program in the text.

*Participants*

We analyzed the elderly registered on SISVAN and monitored on the system, and information on the nutritional status of this population, by consulting consolidated reports, publicly accessible, available in the SISVAN Web platform (https://sisaps.saude.gov.br/sisvan/relatoriopublico/index). For this study, we selected the stage of the life cycle related to the elderly, whose age group includes individuals aged 60 years or older, according to the SISVAN classification.^3^

*Study variables*

Nutritional status, based on BMI, was classified according to the World Health Organization (WHO) recommendation, using the standard formula: weight in kilograms (kg) divided by the square of height in meters (m²). The following BMI cutoff points, specific for older adults, were used in the categorization of this index: underweight (BMI < 22kg/m²); normal weight (BMI between 22kg/m² and 27kg/m²); and overweight (BMI > 27kg/m²).^10^

Regarding correlation analysis, the following continuous variables and their descriptions were used: human development index (HDI), an indicator comprised of data on education, income and life expectancy;^11^ Gini index, used to measure the degree of income concentration;^12^ low individual monthly income [proportion (%) of poor individuals, representing the proportion of people with household income *per capita* below the poverty line]; and low household income [proportion (%) of poor households, representing the proportion of households with household income *per capita* below the poverty line].^13^

The dependent variables were the temporal trends of coverage and distribution of nutritional status categories, and the independent variables corresponded to the region, the reference year and indicators of social inequalities.

*Data source and analysis*

Data were extracted from the SISVAN website and organized in Excel spreadsheet®. The database, its compilation and analysis, and the preparation of tables and graphs were performed using the Power BI platform and the Power BI visualization on a web page.^14^ All records available on the platform were used in the analyses.

The temporal trend of SISVAN coverage was analyzed by calculating the total coverage, represented by the percentage of individuals monitored via SISVAN Web. The percentage of coverage was calculated by dividing the number of records of older people (age ≥ 60 years) with nutritional status information on the SISVAN Web, divided by the population in this same age group defined as SUS users, multiplied by 100.^4^

This calculation was based on data on the total resident population, made available by the Brazilian Institute of Geography and Statistics (*Instituto Brasileiro de Geografia e Estatística* - IBGE),^15^ and the data on the population using the SUS, available from the National Regulatory Agency for Private Health Insurance and Plans (*Agência Nacional de Saúde Suplementar* - ANS).^16^ The same criterion was used by previous studies that evaluated the national coverage of nutritional status and food intake by SISVAN.^4^ ^,^ ^17^

Coverage and prevalence of nutritional status (underweight, normal weight or overweight) were calculated according to the national macro-region, Brazil as a whole and the reference year (independent variable). This information was used to evaluate the temporal change in SISVAN coverage and the distribution of nutritional status categories (dependent variable), at a 95% confidence interval (95%CI).

The temporal trend was analyzed using Prais-Winsten regression models, a recommended approach for ecological studies, to control the self-correction of regression residuals among the years analyzed.^18^ The average annual coverage change and each category of nutritional status were calculated using the following formula:

[-1 + (10β)] x 100

where β is logarithm to base 10, resulting from the Prais-Winsten regression. Non-significant p-values (p ≥ 0.05) indicated a trend of stability, while significant p-values (p < 0.05), indicated rising or decreasing trend, according to positive or negative annual change, respectively.

Correlation coefficients (r) of social inequality indicators (HDI; Gini index) with annual increment rates of nutritional status classifications of older people (underweight; normal weight; overweight) were estimated using Pearson’s correlation test, and p-value < 0.05 was considered significant, with the units of analysis comprised of the 26 Brazilian states and the Federal District.

Analyses of absolute and relative inequalities related to nutritional status were performed, according to the social inequality indicators described, and thus, the slope index of inequality (SII) and the concentration index (CIX) were obtained.^19^ In order to calculate the CIX, the variables HDI, Gini index, GDP *per capita* and number of households and poor individuals were classified into quintiles.

For the significance level, p-value ≤ 0.05 was considered. All statistical analyses were performed using Stata software, version 11.2 (Stata Corp, College Station, TX, USA).

## RESULTS

A total of 11,587,933 records of older adults were identified on SISVAN system during the study period. Between 2008 and 2019, the percentage of SISVAN coverage was less than 3%, nationally and among macro-regions; with the exception of the South region, which showed percentages of coverage greater than 3% in 2017 (3.3%), 2018 (3.9%) and 2019 (5.5%), and the Southeast region, with 3.1% coverage in 2019. It could be seen a statistically significant and marked increase in temporal trend of SISVAN coverage in all macro-regions, with 2019 showing the highest national coverage in all macro-regions (Table 1).

At the national level, the percentage of SISVAN coverage among older adults ranged from 0.1% in 2008 to 2.9% in 2019. The South and Southeast macro-regions presented the highest percentages of coverage in the years analyzed, with the highest value recorded in the South region in 2019 (5.5%). The average annual change for the country (38.4%; 95%CI 28.0;49.7) and for all national macro-regions was positive and statistically significant, showing an increase in the system coverage in the period studied. The lowest annual coverage change was identified in the Midwest (32.2%; 95%CI 21.3;44.3) and Southeast (33.8%; 95%CI 27.7;40.1), while the highest annual change was observed in the North (44.4%; 95%CI 27.9;63.0) and Northeast (45.2%; 95%CI 26.2;66.9) regions (Table 1).

**Table 1:** - Temporal trend of nutritional status coverage of older adults registered on the Food and Nutrition Surveillance System, Brazil, 2008-2019

Brazil and macro-regions	Annual coverage of nutritional status (%)												Annual change (%)^a^	95%CI^b^	p-value	Trend
	2008	2009	2010	2011	2012	2013	2014	2015	2016	2017	2018	2019				
BRAZIL	0.1	0.2	0.2	0.3	0.3	0.3	0.5	1.5	2.0	2.3	2.6	2.9	38.4	28.0;49.7	< 0.001	Rising
North	0.1	0.2	0.1	0.2	0.2	0.2	0.4	1.6	2.1	2.1	2.3	2.5	44.4	27.9;63.0	< 0.001	Rising
Northeast	0.1	0.1	0.1	0.1	0.1	0.1	0.3	1.5	1.8	1.7	1.9	2.2	45.2	26.2;66.9	< 0.001	Rising
South	0.1	0.3	0.3	0.3	0.3	0.3	0.3	1.7	2.6	3.3	3.9	5.5	40.8	22.9;61.9	< 0.001	Rising
Midwest	0.1	0.2	0.2	0.3	0.2	0.3	0.3	1.0	1.4	1.6	2.0	2.1	32.2	21.3;44.3	< 0.001	Rising
Southeast	0.1	0.4	0.3	0.4	0.4	0.6	0.7	1.6	1.9	2.5	2.9	3.1	33.8	27.7;40.1	< 0.001	Rising

a) Annual increment rate, calculated using the formula [-1+(10^β)]×100, in which β is the coefficient resulting from the Prais-Winsten regression; b) 95%CI: 95% confidence interval.

Regarding the classification of nutritional status among older adults registered on SISVAN, an increasing trend in the prevalence of overweight was found at the national level and in all macro-regions. At the national level, overweight among older adults showed a percentage increase of 8.3% in the period from 2008 to 2019, with an annual change of 1.8% (95%CI 1.5;2.2). The Southern region presented the highest percentage of overweight prevalence in all years analyzed, when compared to the other macro-regions. However, the highest annual increase was identified in the North region: 3.1% (Table 2).

**Table 2:** - Temporal trend of the prevalence of overweight in older adults registered on the Food and Nutrition Surveillance System, Brazil, 2008-2019

Brazil and macro-regions	Annual prevalence of overweight (%)												Annual change (%)^a^	95%CI^b^	p-value	Trend
	2008	2009	2010	2011	2012	2013	2014	2015	2016	2017	2018	2019				
BRAZIL	43.1	41.9	43.9	45.0	46.2	45.1	45.2	48.7	48.9	49.7	50.9	51.4	1.8	1.5;2.2	< 0.001	Rising
North	36.9	36.8	39.3	39.6	39.9	42.2	42.6	47.7	47.3	48.0	49.2	49.5	3.1	2.6;3.6	< 0.001	Rising
Northeast	38.9	36.6	40.1	40.3	41.3	38.7	42.5	44.1	44.1	45.0	46.6	46.3	2.1	1.6;2.5	< 0.001	Rising
South	50.5	49.8	50.6	54.2	55.1	56.2	56.2	57.6	55.7	57.1	58.5	58.6	1.5	0.8;2.1	< 0.001	Rising
Midwest	40.9	43.0	43.1	45.1	47.4	46.5	48.3	52.8	52.1	51.3	52.2	52.9	2.4	1.8;3.1	< 0.001	Rising
Southeast	42.1	40.5	43.0	44.1	45.4	44.0	44.4	48.3	49.0	48.7	49.7	50.5	1.9	1.5;2.4	< 0.001	Rising

a) Annual increment rate, calculated using the formula [-1+(10^β)]×100, in which β is the coefficient resulting from the Prais-Winsten regression; b) 95%CI: 95% confidence interval.

On the other hand, the prevalence of underweight showed a decreasing temporal trend in Brazil and in the five macro-regions. At the national level, the percentage of underweight ranged from 18.1% in 2008 to 12.2% in 2019, with a negative annual change of 3.9% (95%CI -4.7;-3.0). Among the macro-regions, the Northeast showed the highest percentage of underweight in all years analyzed, except for the highest results in the North region related to the years 2011 (18.8%) and 2012 (19.0%) (Table 3).

**Table 3:** - Temporal trend of the prevalence of underweight in older adults registered on the Food and Nutrition Surveillance System, Brazil, 2008-2019

Brazil and macro-regions	Annual prevalence of underweight (%)												Annual change (%)^a^	95%CI^b^	p-value	Trend
	2008	2009	2010	2011	2012	2013	2014	2015	2016	2017	2018	2019				
BRAZIL	18.1	18.8	17.3	16.5	15.7	16.6	16.6	13.9	13.6	13.2	12.5	12.2	-3.8	-4.7;-3.0	< 0.001	Decreasing
North	20.1	20.3	18.5	18.8	19.0	17.2	17.5	13.8	14.0	13.4	12.7	12.5	-4.8	-5.9;-3.8	< 0.001	Decreasing
Northeast	20.4	21.6	19.5	18.6	17.7	19.8	17.6	15.6	15.4	14.8	13.9	13.9	-4.0	-4.8;-3.1	< 0.001	Decreasing
South	13.1	13.7	12.6	10.9	10.6	10.6	11.3	9.6	10.5	9.7	8.9	8.9	-3.6	-4.5;-2.6	< 0.001	Decreasing
Midwest	19.2	17.4	17.4	15.6	14.7	15.5	15.2	13.0	12.8	12.8	12.2	11.6	-4.2	-4.8;-3.5	< 0.001	Decreasing
Southeast	19.6	20.0	18.2	17.4	16.4	17.4	17.0	14.4	14.0	14.1	13.5	13.0	-3.9	-4.6;3.1	< 0.001	Decreasing

a) Annual increment rate, calculated using the formula [-1+(10^β)]×100, in which β is the coefficient resulting from the Prais-Winsten regression; b) 95%CI: 95% confidence interval.

It could be seen a decreasing temporal trend in the prevalence of older adults with nutritional status classified as adequate, at the national level and in the five macro-regions. At the national level, the percentage of older adults with nutritional status classified as adequate ranged from 38.7% in 2008 to 36.4% in 2019, representing a negative annual change of 0.7% (95%CI -0.8;-0.5). In all macro-regions, the lowest prevalence of adequate nutritional status was found in 2019, with the exception of the Midwest and Northeast regions, which showed their lowest percentages in 2016 (35.1%) and 2018 (39.6%), respectively (Table 4).

**Table 4:** - Temporal trend of the prevalence of normal weight in older adults registered on the Food and Nutrition Surveillance System, Brazil, 2008-2019

Brazil and macro-regions	Temporal trend of the prevalence of normal weight (%)												Annual change (%)^a^	95%CI^b^	p-value	Trend
	2008	2009	2010	2011	2012	2013	2014	2015	2016	2017	2018	2019				
BRAZIL	38.7	39.4	38.8	38.6	38.1	38.3	38.3	37.5	37.5	37.1	36.6	36.4	-0.7	-0.8;-0.5	< 0.001	Decreasing
North	43.0	42.9	42.2	41.6	41.2	40.5	39.9	38.5	38.7	38.5	38.1	38.1	-1.2	-1.5;-1.0	< 0.001	Decreasing
Northeast	40.7	41.8	40.4	41.1	41.0	41.5	40.0	40.3	40.5	40.2	39.6	39.8	-0.4	-0.5;-0.2	0.001	Decreasing
South	36.4	36.5	36.8	35.0	34.3	33.2	32.5	32.8	33.8	33.2	32.6	32.5	-1.1	-1.8;-0.5	0.004	Decreasing
Midwest	39.9	39.7	39.5	39.3	37.9	38.0	36.5	34.3	35.1	35.9	35.6	35.5	-1.2	-1.9;-0.6	0.002	Decreasing
Southeast	38.3	39.5	38.8	38.5	38.2	38.6	38.6	37.3	37.0	37.2	36.8	36.5	-0.6	-0.8;-0.4	< 0.001	Decreasing

a) Annual increment rate, calculated using the formula [-1+(10^β)]×100, in which β is the coefficient resulting from the Prais-Winsten regression; b) 95%CI: 95% confidence interval.

With regard to the social inequality indicators analyzed in the correlation with the annual increment rate according to nutritional status, only HDI and GDP *per capita* presented statistically significant correlation coefficient (r). A moderate positive correlation with annual increment rate of underweight between HDI (p-value = 0.003; r = 0.556) and GDP *per capita* (p-value < 0.001; r = 0.681) was found. A moderate inverse correlation with annual increment rate of overweight between HDI (p-value = 0.002; r = -0.565) and GDP *per capita* (p-value = 0.007; r = -0.508) was found.

The analyses of slope index of inequality confirmed the correlation between HDI, GDP *per capita* and nutritional status of older adults (underweight, normal weight or overweight). Regarding underweight, the slope index values were positive for HDI (p-value = 0.003) and GDP *per capita* (p-value < 0.001). As for overweight, the values were negative for HDI (p-value = 0.002) and GDP *per capita* (p-value = 0.007). The analyses of concentration index presented negative values, that is, the corresponding concentration curve lies above the diagonal, showing that the least favored FUs (in relation to HDI and GDP *per capita*) accumulated the highest rates of underweight and overweight when compared to the FUs with the highest values of HDI and GDP *per capita* (p-value < 0.05) (Table 5).

**Table 5:** - Absolute and relative inequalities in nutritional status according to indicators of social inequality in older adults registered on the Food and Nutrition Surveillance System, Brazil, 2008-2019

Indicators of social inequality	SII: slope index of inequality^a^		
	Underweight	Normal weight	Overweight
Human Development Index (HDI)	26.77 (0.003)	3.80 (0.372)	-17.48 (0.002)
Gini index	15.98 (0.108)	7.44 (0.090)	-6.36 (0.327)
Gross domestic product (GDP) *per capita*	0.0001 (<0.001)	0.000 (0.448)	-0.0001 (0.007)
Low individual monthly income (proportion of poor people)	-0.07 (0.075)	-0.001 (0.930)	0.05 (0.058)
Low household income (proportion of poor households)	-0.09 (0.066)	0.001 (0.981)	0.06 (0.056)
	CIX: concentration index^a^		
	Underweight	Normal weight	Overweight
Human Development Index (HDI)	-0.15 (0.018)	-0.05 (0.672)	-0.19 (0.007)
Gini index	-0.08 (0.184)	-0.14 (0.242)	-0.06 (0.394)
Gross domestic product (GDP) *per capita*	-0.14 (0.024)	0.02 (0.871)	-0.15 (0.040)
Low individual monthly income (proportion of poor people)	0.11 (0.098)	0.05 (0.676)	0.13 (0.070)
Low household income (proportion of poor households)	0.12 (0.083)	0.05 (0.670)	0.13 (0.068)

a) Statistically significant index values (p < 0.05).

## DISCUSSION

It could be seen a low coverage of the nutritional status of older adults by SISVAN, with a rising and significant temporal trend at the national level and in the five national macro-regions. Regarding the classification of nutritional status, there was an increase in the prevalence of overweight, which was inversely related to HDI and GDP *per capita*, at the same time there was a decreasing temporal trend in the prevalence of normal weight and underweight, the latter directly related to HDI and GDP *per capita*, at national level and in the five macro-regions. The analyses of concentration index showed negative values, demonstrating that the least favored FUs (in relation to HDI and GDP *per capita*) accumulated highest increment rates of underweight and overweight in relation to the FUs with the highest values of HDI and GDP *per capita*.

The low coverage values found and the lack of knowledge about the criteria that define the proportions of coverage verified, lead to the observation that estimates of prevalence of nutritional status are limited to the group of people covered by SISVAN, and it is not recommended to extrapolate these data to the general population. The maternal-infant population has been the greatest representativeness of SISVAN, since the implementation of its online version, given that the conditionalities of the Bolsa Família Program do not include the follow-up of adults and older adults.^4^ It seems seriously compromising, in terms of reliability, that a nationwide system, aimed at monitoring the nutritional status of the Brazilian population, does not offer data that can be read as representative of the general population. However, this point highlights the estimated rates of positive change for coverage estimates throughout the country and its macro-regions, pointing to the mobilization and directing efforts of teams from local health networks in order to put the proposal for surveillance of nutritional status of the older adults into practice. Despite the difficulties, SISVAN has been constantly strengthened and expanded, having as one of its main challenges entering data and the incorporation of the system itself into the routine primary health care services, which is responsibility of managers and health professionals.

The low coverage of the nutritional status of older adults by SISVAN, with a significant increasing trend in coverage, was identified in an analysis performed using stratification of life cycles, during the first six years of implementation of SISVAN Web, from 2008 to 2013.^4^ The results of this study confirm the increasing in coverage expected for the age group of 60 years and older. Nevertheless, the data found remain below the data expected for this group, when the predominance of follow-up of other stages of life is observed, such as those of children, adolescents and pregnant women. For example, over a five-year period (2008-2012), the national coverage of the nutritional status of preschoolers by SISVAN ranged from 17.7% to 27.9%, while that of older adults ranged from only 0.4% to 1.2% during the same period.^4^

The low coverage of the nutritional status of older adults by SISVAN is worrisome, because they are the fastest growing segment of the Brazilian population due to the process of demographic transition, caused, among other factors, by declining birth rates and increased life expectancy.^20^ Along with rapid population aging, the epidemiological transition is taking place, changing the profile of morbidity and mortality. As such, we could say that the Brazilian elderly population lives longer, but not necessarily better. At the age of 60, the emergence of chronic non-communicable diseases (NCDs) and their consequences are more evident, and may reduce or hinder the independence and autonomy of people in this age group.^21^

The prevalence of normal weight showed the lowest annual change observed in the country, with a decreasing trend. As mentioned before, the coverage verified in the period is so low that it is impossible to extrapolate the estimated prevalence for the total population considered in the study. However, a decreasing trend in the prevalence of normal weight is even more worrisome, if we take into consideration that the aging process can cause changes in body composition of older adults, including an increase and redistribution of fat mass and concomitant reduction of lean mass and bone density, factors that are independent of changes in body weight and BMI.^21^

Regional differences in the distribution of nutritional status classification of older adults were also observed in a study that evaluated individuals aged 60 years or older, taking part in the 2008-2009 Family Budget Survey (Pesquisa de Orçamentos Familiares - POF), conducted by IBGE. A higher prevalence of underweight was found in residents of rural areas and in the Northeast and Midwest regions.^12^ Underweight has historically been related to socioeconomic problem in Brazil^23^ and, in this study, its annual increment rate was directly related to HDI and GDP *per capita* of the FUs. These results, however, should be interpreted with caution, given that the low coverage of the nutritional status of older adults may have interfered with this correlation. However, the analyses of concentration index found that the FUs with the worst socioeconomic profile had the highest increment rates of underweight and overweight.

Indicators of social inequality are often directly associated with better living conditions. According to a systematic review, HDI was directly related to a better gait speed (considered a marker of overall health status in older adults), suggesting that education, income and life expectancy affect this marker performance.^24^ Similarly, GDP growth in China was associated with greater physical fitness of the elderly, a result attributed to increased financial investments in public sports and health services in that country.^25^

Unexpectedly, the increment rate of overweight was inversely related to HDI and GDP *per capita* of the Brazilian FUs. According to another systematic review, dedicated to investigating the nutritional status of older adults in Africa, overweight was positively related to HDI, with a higher prevalence found in countries with better socioeconomic conditions.^26^ In Brazil, overweight is more prevalent in older adults living in the South and Southeast regions,^11^ a fact generally attributed to the economic and social differences historically present in the configuration of Brazilian regions, which include inequalities in income, schooling, basic sanitation and housing conditions, with South and Southeast regions showing better rates. These socioeconomic inequalities influence the availability of and access to goods and services, affecting the quality of life and health conditions of the general population.^27^

However, it is worth considering that the greatest change in coverage proportions on SISVAN occurred in the North and Northeast regions; in turn, the highest coverage observed corresponded exactly to the Southern region, in the last year analyzed, and it is possible that the different proportions of coverage in the system are also related to trends in nutritional status indicators, which would explain the highest prevalence of overweight in Southern Brazil. A greater detection of overweight may possibly be related to a greater search for people with this profile by health services, or to a greater demand for health services for these people. Therefore, there may be some selection bias in the generation of the estimated data.

The increase in the prevalence of overweight and the decreasing trend of underweight and normal weight may indicate the nutritional transition for the older adult population, as initially verified among the adult population. This process, which has been underway for 40 years in the country,^28^ is characterized by a decrease in the prevalence of malnutrition and an increase in the occurrence of overweight. Initially, a higher prevalence of overweight and obesity was observed in Brazilian regions with the best socioeconomic status.^23^ In the last decade, the occurrence of overweight has been increasing among the low-income adult population.^28^ This fact was observed in the increase in the prevalence of overweight among older adults followed by SISVAN at the national level, taking into consideration that most of the population using the SUS has lower income, when compared to people with private health insurance plans.^29^ This finding can help clarify the inverse correlations between the annual increment rate of overweight and the indicators of social inequality - HDI and GDP *per capita*, found in this study.

Despite the increasing trend in the coverage found, SISVAN has not been used to its full potential since the creation of its web platform. Although there is collection and entry of weight and height information, those responsible for the system, in general, do not use the data generated for the planning, management and evaluation of food and nutrition actions.^30^ Some of the main reasons given for this gap include the complexity of the system, professional training and work overload.^4^

It is noteworthy that the results of this study do not allow conclusions at the individual level, since it is an investigation of ecological and aggregate analysis, which is one of the factors capable of interfering in the interpretation of the findings. The use of secondary data is also a restriction, because they come from different sources and, consequently, inconstancy in the credibility of the information, which is susceptible to errors during the collection, typing and under-recording, among others. However, given the lack of studies on the subject, these findings can contribute to generate more hypotheses about the relationship between social inequalities and the nutritional status of older adults.

The low percentage of coverage, with a significant increasing trend for the older adult population, observed in the first 12 years of SISVAN, indicates that its use is in an adaptation process, resulting in the production of insufficient data to support the development and adjustment of public policies for the prevention of diseases/health conditions, as well as health promotion and maintenance aimed at this population. And, in view of the new needs of nutritional care identified by demographic and epidemiological transitions, the increase in coverage of Food and Nutrition Surveillance in this stage of the life cycle is also characterized as preferential and indispensable for planning health actions for the older adult population.

Regional inequalities were identified in the distribution of nutritional status classifications, with the highest rates of overweight in the South region, while the highest rates of underweight were found in the North and Northeast regions. The increasing trend in the prevalence of overweight and the decrease in the occurrence of underweight and normal weight in all Brazilian macro-regions suggest the occurrence of the nutritional transition process for the older adult population, similarly to what was identified for the Brazilian adult population in the 1970s, 1980s and 1990s.^23^

It could be seen the need to incorporate the actions of SISVAN into the routine primary health care services, as a way to boost the coverage of the system. Therefore, it is essential to raise awareness among professionals and managers about the importance of data collection and use of information, in addition to the structural support and use of the Brazilian SIVAN, for the situational diagnosis of food and nutrition in all stages of the life cycle. Such actions can positively impact the coverage and data quality, benefiting the population through effective monitoring of their nutritional health.
