# Erratum

**DOI:** 10.1590/S2237-96222023000100033

**Published:** 2023-05-08

In the article **“Food and Nutrition Surveillance System (SISVAN) coverage, nutritional status of older adults and its relationship with social inequalities in Brazil, 2008-2019: an ecological time-series study”**, published on Epidemiology and Health Services, 32(1):e2022595, 2023, in the page 1:

Original text:

10.1590/S2237-96222023000100003

Corrected text:

10.1590/S2237-96222023000100034

